# *Escherichia ruysiae* sp. nov., a novel Gram-stain-negative bacterium, isolated from a faecal sample of an international traveller

**DOI:** 10.1099/ijsem.0.004609

**Published:** 2021-01-06

**Authors:** Boas C. L. van der Putten, S. Matamoros, D. R. Mende, E. R. Scholl, COMBAT consortium†, C. Schultsz

**Affiliations:** ^1^​ Department of Medical Microbiology, Amsterdam UMC, University of Amsterdam, Amsterdam, the Netherlands; ^2^​ Department of Global Health, Amsterdam Institute for Global Health and Development, Amsterdam UMC, University of Amsterdam, Amsterdam, the Netherlands; ^3^​ Electron Microscopy Center Amsterdam, Amsterdam UMC, University of Amsterdam, Amsterdam, the Netherlands

**Keywords:** bacterial taxonomy, Enterobacteriaceae, *Escherichia*

## Abstract

The genus *Escherichia* comprises five species and at least five lineages currently not assigned to any species, termed ‘* Escherichia* cryptic clades’. We isolated an *Escherichia* strain from an international traveller and resolved the complete DNA sequence of the chromosome and an IncI multidrug resistance plasmid using Illumina and Nanopore whole-genome sequencing (WGS). Strain OPT1704^T^ can be differentiated from existing *Escherichia* species using biochemical (VITEK2) and genomic tests [average nucleotide identity (ANI) and digital DNA–DNA hybridization (dDDH)]. Phylogenetic analysis based on alignment of 16S rRNA sequences and 682 concatenated core genes showed similar results. Our analysis further revealed that strain OPT1704^T^ falls within *Escherichia* cryptic clade IV and is closely related to cryptic clade III. Combining our analyses with publicly available WGS data of cryptic clades III and IV from Enterobase confirmed the close relationship between clades III and IV (>96 % interclade ANI), warranting assignment of both clades to the same novel species. We propose *Escherichia ruysiae* sp. nov. as a novel species, encompassing *Escherichia* cryptic clades III and IV (type strain OPT1704^T^=NCCB 100732^T^=NCTC 14359^T^).

## Introduction

Within the genus *Escherichia *, five species are recognized: *Escherichia coli* [1], *Escherichia hermannii* [2], *Escherichia fergusonii* [3], *Escherichia albertii* [4] and, most recently, *Escherichia marmotae* [5]. It has been proposed to reassign *E. hermannii* to *Atlantibacter hermannii* [6], but this reassignment is not yet formally approved. Several other species were previously reassigned from the genus *Escherichia* to other genera, such as *E. vulneris* (now *Pseudescherichia vulneris* [7]), *E. blattae* (now *Shimwellia blattae* [8]) and *E. adecarboxylata* (now *Leclercia adecarboxylata* [9]). All five current *Escherichia* species have been associated with the potential to cause animal and/or human disease [2, 10–13]. Several *Escherichia* strains cannot be assigned to any of the five existing species [14]. Based on analysis of genomic data, these strains cluster into several groups, which were termed ‘* Escherichia* cryptic clades’, numbered I through VI [14, 15]. Recently, cryptic clade V was formally recognized as a separate species (* E. marmotae *), leaving at least five cryptic clades that have not been delineated at the species level [5]. Here we report the novel species, *Escherichia ruysiae* sp. nov., isolated from faecal material of an international traveller. *Escherichia ruysiae* sp. nov. encompasses the closely related *Escherichia* cryptic clades III and IV.

## Isolation and ecology

We discovered a cryptic clade IV strain in our collection, previously identified as extended-spectrum β-lactamase (ESBL)-producing *E. coli* as part of the combat study, which investigated the acquisition of ESBL-producing Enterobacteriaceae (ESBL-E) during international travel [16]. This isolate, OPT1704^T^, was further characterized in detail.

The strain was isolated from a human faecal sample provided immediately after an individual’s return from a 1 month journey to several Asian countries. No ESBL-E were detected in a faecal sample collected immediately before departure, suggesting that the ESBL gene, and possibly strain OPT1704^T^, were acquired during travel. The traveller reported diarrhoea during travel but no antibiotic usage. No ESBL-E were isolated in follow-up faecal samples, suggesting loss of the OPT1704^T^ strain or the ESBL gene within 1 month after return from travel.

## Genome features

The whole-genome sequence of strain OPT1704^T^ was determined using a combination of the Illumina HiSeq and Oxford Nanopore Technologies (ONT) sequencing platforms. Strain OPT1704^T^ was grown in liquid lysogeny broth (LB) at 37 °C. DNA for Illumina sequencing was extracted using the Qiagen Blood and Tissue kit (cat. no. 69 506, Qiagen) and the sequencing library was prepared using the Illumina Nextera XT DNA Library Preparation kit (cat. no. FC-131–1096, Illumina), both according to manufacturer’s instructions. DNA for ONT sequencing was extracted using the Qiagen MagAttract HMW DNA extraction kit (cat. no. 67 563, Qiagen) and the sequencing library was prepared using the native barcoding and ligation sequencing kits (cat. nos. EXP-NBD114 and SQK-LSK109, respectively, ONT) according to manufacturer’s instructions. The Illumina sequencing run yielded a total of 6.3×10^6^ paired-end reads, with a mean read length of 151 bp. Default parameters were used in bioinformatic analyses unless noted otherwise. Illumina reads were filtered using fastp (using flag ‘--disable-length-filtering’, version 0.19.5 [17]) and downsampled using seqtk (version 1.3-r106, https://github.com/lh3/seqtk) to provide a theoretical coverage depth of 100× with the assumption that strain OPT1704^T^ has a genome size of approximately 5×10^6^ bp. The ONT sequencing run yielded a total of 2.5×10^4^ reads, with a mean read length of 9078 bp before filtering. ONT reads were filtered on length and on read identity using Filtlong (version 0.2.0, https://github.com/rrwick/Filtlong) with Illumina reads as a reference, leaving 1.5×10^4^ reads with a mean length of 12 580 bp. This provided a theoretical coverage depth of ~38× of ONT reads. The combined assembly using Unicycler (version 0.4.6 [18]) of Illumina and Nanopore reads resulted in a completely assembled genome, consisting of one circular chromosome (4 651 588 bp) and one circular plasmid (116 086 bp). The G+C content of the complete strain OPT1704^T^ genome was 50.6 mol%.

Putative resistance and virulence genes were predicted from the complete genome using ABRicate (https://github.com/tseemann/abricate) with the CARD [19] and VFDB [20] databases. Strain OPT1704^T^ harbours six resistance genes on its IncI plasmid, associated with reduced susceptibility to fluoroquinolones (*qnrS1*), aminoglycosides (*aph [6]-Id* and *aph(3′′)-Ib*), cephalosporins (*blaCTX-M-14*), trimethoprim (*dfrA14*) and sulphonamides (*sul2*), corresponding with its reduced susceptibility to fluoroquinolones (norfloxacin, MIC: 2 mg l^−1^ and ciprofloxacin, MIC: 0.5 mg l^−1^), cephalosporins (cefuroxime, MIC: >32 mg l^−1^ and cefotaxime, MIC: 4 mg l^−1^) and trimethoprim–sulfamethoxazole (MIC: >8 mg l^−1^), assessed using VITEK2 (bioMérieux). However, strain OPT1704^T^ was susceptible to tobramycin (MIC: ≤1 mg l^−1^) and gentamicin (MIC: ≤1 mg l^−1^) despite the presence of aminoglycoside resistance genes *aph(6)-Id* and *aph(3′′)-Ib*. The *aph(6)-Id* gene encodes an aminoglycoside-modifying enzyme that mediates resistance against streptomycin [21]. The *aph(3′′)-Ib* gene encodes an aminoglycoside-modifying enzyme mediating resistance against tobramycin and gentamicin [22] but the *aph(3′′)-Ib* variant identified in strain OPT1704^T^ possesses a Glu18Lys mutation which maps to the catalytic phosphorylase kinase domain (assessed with InterPro [23]). This could potentially inhibit enzymatic function, explaining the observed susceptibility to gentamicin and tobramycin, based on clinical breakpoints [24]. Furthermore, several putative virulence genes were predicted from the genome sequence associated with siderophore function (*chuX*, *entS*, *fepABD*), fimbriae (*fimBCDGI*), a type II secretion system (*gspGHI*) and capsular polysaccharide biosynthesis (*kpsD*). These predicted virulence genes, when present in *Escherichia coli *, are not typically associated with a specific clinical syndrome such as diarrhoeal disease.

## Physiology and chemotaxonomy

Strain OPT1704^T^ formed circular, grey-white colonies on a Columbia sheep (COS) blood agar plate when incubated overnight at 37 °C. No haemolysis was observed. Individual cells were observed using TEM and were rod-shaped and on average 0.7×1.9 µm in size (Fig. S1, available in the online version of this article). Bacteria were fixed with McDowell fixative (4 % v/v PFA and 1 % v/v GA in 0.1 M phosphate buffer) with 1.5 % lysine acetate (Merck) for 4 h and postfixed with 1 % osmium tetraoxide (Electron Microscopy Sciences) for 1 h. Afterwards, the bacteria were dehydrated using an ethanol series and embedded in Epon 812 (Ladd Research). Copper grids covered with formvar were used to collect 60–70 nm sections made using a Leica EM FC6 ultramicrotome. Sections were stained with uranyl acetate (Merck) and lead citrate (Laurylab). Electron micrographs were collected using an FEI Tecnai T12 Biotwin electron microscope operated at 120 kV and equipped with an EMSIS Xarosa camera. Subsequently, we tested motility using the hanging-drop method, oxidase presence using an oxidase strip (cat. no. 40 560, Sigma Aldrich) and catalase presence using H_2_O_2_ [25]. The strain was shown to be Gram-stain-negative, non-motile, oxidase-negative and catalase-positive. The strain was capable to grow in the absence of oxygen. On COS blood plates, it showed growth in the temperature range of 20–42 °C. The strain was also able to grow in NaCl concentrations ranging from 0–6 % w/v in lysogeny broth overnight at 37 ˚C, but not at NaCl concentrations from 7–10 % w/v (1 % steps). MALDI-TOF (Bruker) and VITEK2 (BioMérieux) systems both identified strain OPT1704^T^ as *E. coli* with high confidence scores (score >2 for MALDI-TOF and ‘Excellent identification’ for VITEK2, see Supplemental information for MALDI-TOF spectrum). Comparison of the output of the VITEK2 biochemical test with published biochemical reactions of other *Escherichia* species revealed that *E. ruysiae* sp. nov. OPT1704^T^ is distinct from other *Escherichia* species based on a combination of biochemical markers (Table 1) [2–5, 26].

**Table 1.** Comparison of biochemical markers which differentiate *E. ruysiae* sp. nov. from other *Escherichia* species Data for *E. albertii, E. coli, E. fergusonii* and *E. marmotae* summarised from literature [2–5, 26]. + and – indicate that ≥85 % of tested strains is positive or negative for that biochemical marker, respectively.

	* E. ruysiae *	* E. albertii *	* E. coli *	* E. fergusonii *	* E. hermannii *	* E. marmotae *
ONPG	+	+	+	+	+	−
Lysine decarboxylase	−	+	+	+	−	+
Ornithine decarboxylase	+	+	+*	+	+	−
Fermentation of:						
Adonitol	−	−	−	+	−	−
d-Xylose	−	−	+	+	+	+
Cellobiose	−	−	−	+	+	−
d-Sorbitol	+	−	+	−	−	+

*50–85 % of *E. coli* possess this biochemical property.

## 16s rRNA gene and whole-genome phylogeny

Next, we calculated 16S rRNA sequence similarities, average nucleotide identity (ANI) values and digital DNA–DNA hybridization (dDDH) values between strain OPT1704^T^ and type strains of the other *Escherichia* species, representative genomes of the other three *Escherichia* cryptic clades and *S. enterica* serovar Typhimurium (Table 2). Representative genomes for the *Escherichia* cryptic clades I, II, III and VI were selected from Enterobase [27], using the genomes with the highest contiguity. Clades VII and VIII in Enterobase only consisted of a single strain and were not used in further analyses. We used three separate tools to calculate ANI (fastANI [28], OrthoANIu [29] and ANI calculator from Enveomics [30]). Multiple ANI calculation algorithms were employed to increase confidence in the genomic species delineation, as different ANI algorithms can output different ANI values. In this study, calculated ANI values were similar across ANI calculation algorithms. We also included calculation of the dDDH values between strains using the DSMZ Genome-to-Genome Distance Calculator [31]. The output of formula 2 of the DSMZ Genome-to-Genome Distance Calculator was used, as recommended by the authors of the tool. 16S rRNA genes were extracted from whole genomes using barrnap (version 0.9, https://github.com/tseemann/barrnap) and single nucleotide polymorphisms (SNPs) between strains were counted using snp-dists (version 0.6, https://github.com/tseemann/snp-dists). Extracted 16S rRNA gene segments were 1538 bp long for all strains and were manually aligned and checked. The alignment is provided in the supplementary material.

**Table 2.** Comparison of strain OPT1704^T^ 16S rRNA and whole-genome sequence with type strains of *E. albertii *, *E. coli *, *E. fergusonii *, *E. marmotae *, representative genomes of *Escherichia* cryptic clades I, II, III and VI and *S. enterica* serovar Typhimurium In bold are the values that warrant assignment of strain OPT1704^T^ to a novel species (<98.7 % 16S rRNA sequence similarity, <95–96 % ANI, <70 % dDDH). ANI, average nucleotide identity; dDDH, digital DNA–DNA hybridization.

	* E. ruysiae* sp. nov. OPT1704^T^				
	16S rRNA sequence similarity (%)	ANI (%, fastANI)	ANI (%, OrthoANIu)	ANI (%, ANI calculator Enveomics)	dDDH (%)
* E. albertii * NBRC 107761^T^	**98.6**	**90.0**	**90.0**	**89.2**	**39.8**
* E. coli * ATCC 11775^T^	98.7	**92.8**	**92.4**	**92.0**	**48.3**
* E. fergusonii * ATCC 35469^T^	98.9	**89.4**	**88.2**	**89.7**	**36.7**
*E. hermanni* NCTC 12129^T^	**98.0**	**80.2**	**77.7**	**80.1**	**21.4**
* E. marmotae * HT073016^T^	98.9	**92.2**	**92.2**	**91.4**	**47.1**
* S. enterica * Typhimurium LT2^T^	**97.5**	**82.1**	**80.7**	**81.8**	**24.0**
* Escherichia* cryptic clade I 89–3506	99.0	**92.5**	**92.1**	**91.8**	**47.8**
* Escherichia* cryptic clade II MOD1-EC7253	99.2	**92.0**	**91.7**	**91.0**	**45.5**
* Escherichia* cryptic clade III E4694	99.7	96.6	96.5	96.3	70.8
* Escherichia* cryptic clade VI UHCL_3L	**98.4**	**91.6**	**91.7**	**91.3**	**45.9**

Strain OPT1704^T^ showed 98.7–98.9 % 16S rRNA sequence similarity to *E. coli* ATCC 11775^T^, *E. fergusonii* ATCC 35469^T^ and *E. marmotae* HT073016^T^, which would not warrant assignment to a novel species based on the current threshold for species delineation (less than 98.7 % sequence similarity [32]). However, the threshold for species delineation on the basis of 16S rRNA sequence has changed often and thresholds of up to 99 % sequence similarity have been proposed previously [33]. In contrast, ANI analysis and dDDH did support assignment of strain OPT1704^T^ to a novel species, together with the representative strain of *Escherichia* cryptic clade III (Table 2). The analyses also suggested that strain OPT1704^T^ falls within the genus *Escherichia *. This novel species, encompassing both *Escherichia* cryptic clades III and IV, was assigned *E. ruysiae* sp. nov. with strain OPT1704^T^ as the proposed type strain.

Assigning a novel species to a particular genus is challenging and currently no clear guidelines exist. Several approaches have been proposed, such as phylogenomics [32] or counting shared genes [34]. Our phylogenomic analyses show OPT1704^T^ clusters closely with other *Escherichia* species, and clusters further away from *E. hermannii* and *S. enterica *. This clustering pattern is well supported by the bootstrapping analysis, for both the alignment 16S rRNA genes and 682 concatenated core genes. Another commonly used approach is calculating the percentage of conserved proteins (POCP) [34]. Strain OPT1704^T^ had the highest POCP with organisms in the genus *Escherichia *, clearly above the ‘universal’ cutoff of 50 % shared proteins. This cutoff seems to be inadequate for Enterobacterales: *Salmonella enterica* and *Escherichia hermannii* showed a POCP of more than 60 % with all true *Escherichia* strains (Table S1). As the original POCP approach was developed using only 17 genera and has not been verified for Enterobacterales, the high rate of genetic exchange in this order [35] may necessitate an alternative POCP cutoff to the previously proposed cutoff of 50 %.

**Fig. 1.**
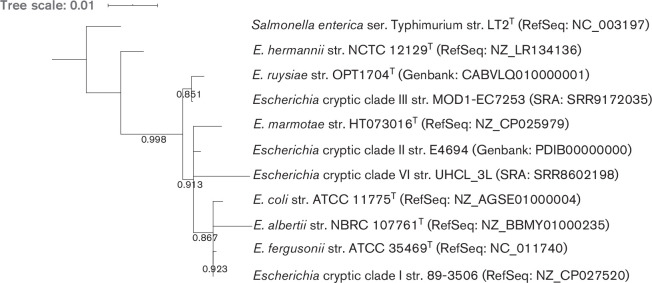
16S phylogeny of *Escherichia ruysiae* sp. nov. OPT1704^T^ with type strains of other *Escherichia* species, other *Escherichia* cryptic clades and *Salmonella enterica* serovar Typhimurium as the outgroup. Numbers indicate bootstraps on a scale of 0 to 1. Phylogeny available at https://itol.embl.de/tree/14511722611226771596704407.

**Fig. 2.**
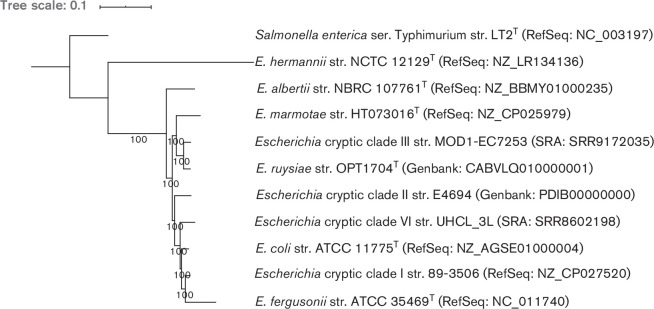
Phylogeny based on 682 concatenated core genes including *Escherichia ruysiae* OPT1704^T^ with type strains of other *Escherichia* species, other *Escherichia* cryptic clades and *Salmonella enterica* serovar Typhimurium as outgroup. Numbers indicate bootstraps on a scale of 0 to 100. Phylogeny available at https://itol.embl.de/tree/14511722611160731596708541.

To gain a better understanding of the genus *Escherichia *, we produced two phylogenies, based on 16S rRNA sequence (Fig. 1) and on an alignment of 682 core genes (Fig. 2). In short, rRNA genes were predicted from whole genomes using barrnap (version 0.9, https://github.com/tseemann/barrnap) and a tree was generated using FastTree (version 2.1.10 [36]). For the core gene alignment, genomes were first annotated with Prokka (version 1.14.0 [37]) and a core gene alignment was produced using Roary (version 3.12.0 [38]) and mafft (version 7.307 [39]). The phylogeny was inferred using a generalised time reversible model using base frequencies from the SNP alignment and free rate heterogeneity (GTR+F+R4 model) in IQ-tree (version 1.6.6 [40]), as advised by ModelFinder [41]. Phylogenies were rooted on the *Salmonella enterica* serovar Typhimurium strain LT2^T^ genome. Both phylogenies showed that strain OPT1704^T^ clusters closely with the strain MOD1-EC7259 from *Escherichia* cryptic clade III, and away from the current *Escherichia* species. Comparing the number of SNPs extracted from the core genome of the strains included in Table 2 showed the same results, as the two strains with the smallest number of SNPs were strains OPT1704T (clade IV) and MOD1-EC7259 (clade III, Table S2).

Chun *et al*. [32] proposed that strains with >95–96 % genome-wide ANI between each other should be assigned to the same species. If cryptic clades III and clade IV would share >95–96 % ANI, this would mean both clades should be assigned to the same novel species, *E. ruysiae* sp. nov. To assess this for a larger number of strains than the type strains presented in Table 2, we downloaded all 65 available WGS from clade III and clade IV strains from Enterobase and compared ANI between all genomes using fastANI (version 1.1 [28]). This analysis revealed that within 33 clade III genomes, the median ANI is 98.6 % (range: 97.7–99.9 %), while within 32 clade IV genomes, the median ANI is 98.9 % (range: 98.6–99.9 %; Table S3). Between clade III and clade IV genomes, the median ANI is 96.5 % (range: 96.1–96.8 %). This suggests clades III and IV should be assigned to the same novel species, *E. ruysiae* sp. nov. Subsequently, based on ANI analysis we selected 10 representative clade IV genomes and 10 representative clade III genomes to be analysed using the TYGS platform [42]. The TYGS platform employs dDDH estimation and 16S and core genome phylogenetics to define species and subspecies within a given set of genomes, with an upload limit of 20 user-provided genomes. The TYGS analysis also indicated that clade III and clade IV should be assigned to a single species, but could be delineated into two separate subspecies (table S4). Currently, no IJSEM guidelines exist for the delineation of subspecies based on genomic data. However, *E. ruysiae* sp. nov. could potentially be delineated further into two subspecies (representing the current clades III and IV, respectively) in the future, after a type strain for cryptic clade III has been identified. In the meantime, we propose to term clades III and IV genomic lineages of *E. ruysiae* sp. nov.

Finally, we annotated the genomes of the strains provided in Table 2 using the EggNOG database [43] and extracted categories of cluster of orthologous genes (COG categories). Strain OPT1704^T^ did not encode a different profile of COG categories compared to other *Escherichia* type strains (Table S5). Possibly, a gene ontology analysis which includes more genomes from all species might elucidate the different functional profiles, but this is out of the scope of the current study.

Based on phenotypic and genotypic data presented above, the niche for *E. ruysiae* sp. nov. cannot be exactly defined yet. Although OPT1704^T^ was isolated from human faeces, earlier studies have indicated that strains belonging to *E. ruysiae* sp. nov. do not adhere well to human-derived cell lines [44]. This finding is highlighted by the fact that we could not detect OPT1704^T^ anymore using ESBL microarray a month after we first detected it, although this might also be caused by loss of the ESBL gene. In conclusion, it seems that the human gut is not the primary niche for *E. ruysiae* sp. nov.

## Description of *Escherichia ruysiae* sp. nov.

*Escherichia ruysiae* (ruy′si.ae N.L. gen. n. *ruysiae* named after Anna Charlotte Ruys, Professor of Microbiology at the University of Amsterdam from 1940 to 1969).

Cells are Gram-stain-negative, facultatively anaerobic, non-sporulating, non-motile rods with a size of approximately 1×2 µm. Colonies are circular, convex, grey-white and semi-transparent when grown overnight at 37 °C on COS agar plates. The species is catalase-positive and oxidase-negative and grows at temperatures between 20 and 42 °C and NaCl concentrations between 0 and 6 % w/v. In the VITEK2 GN biochemical test set it yields positive results for β-galactosidase, d-glucose, maltose, d-mannitol, d-mannose, d-sorbitol, trehalose, sucrose, d-tagatose, γ-glutamyl-transferase, fermentation glucose, tyrosine arylamidase, succinate alkalinization, α-galactosidase, ornithine decarboxylase, courmarate, β-glucoronidase, 0/129 resistance (Comp. Vibrio.) and Ellman and negative for Ala-Phe-Pro-Arylamidase, adonitol, l-pyrrolydonyl-arylamidase, l-arabitol, cellobiose, H_2_S production, β-*N*-acetyl glucosaminidase, glutamyl arylamidase Pna, β-glucosidase, β-xylosidase, β-alanine arylamidase Pna, l-proline arylamidase, lipase, palatinose, urease, citrate (sodium), malonate, 5-keto-d-gluconate, l-lactate alkalinization, α-glucosidase, β-*N*-acetyl-galactosaminidase, phosphatase, glycine arylamidase, lysine decarboxylase, l-histidine assimilation, Glu-Gly-Arg-arylamidase, l-malate assimilation and l-lactate assimilation (Table S6).

The type strain, OPT1704^T^ (=NCCB 100732^T^=NCTC 14359^T^), was isolated from faecal material of an international traveller returning from Asia.

The 16S rRNA sequence is deposited in ENA under accession LR745848. Raw Illumina and Nanopore whole-genome sequencing data, as well as the complete genome assembly are deposited under project PRJEB34275.

## Supplementary Data

Supplementary material 1

Supplementary material 2
